# Viscoelastic Properties of Produced Water Emulsions

**DOI:** 10.1021/acs.energyfuels.6c01394

**Published:** 2026-06-23

**Authors:** Alireza Zahedi, Saeed Azizi, Mark Krzmarzick, Clint P. Aichele

**Affiliations:** † School of Chemical Engineering, 7618 Oklahoma State University, Stillwater, Oklahoma 74078, United States; ‡ School of Civil and Environmental Engineering, Oklahoma State University, Stillwater, Oklahoma 74078, United States

## Abstract

Understanding viscoelastic properties of fluid interfaces leads to improved oil–water separation strategies. This study provides interfacial tension and dilatational viscoelasticity data for cyclohexane in contact with synthetic produced water (PW) or DI water containing the anionic surfactant sodium dodecyl sulfate (SDS). Pendant drop tensiometry with oscillatory dilatational rheology was applied over SDS concentrations of 0.001–1 g/L and frequencies of 0.1–0.5 Hz. The PW–cyclohexane system exhibited interfacial tension (44.4 ± 0.32 mN/m) lower than that of the corresponding DI water–cyclohexane interface (47.9 ± 1.26). The apparent critical micelle concentration (CMC) at the PW–cyclohexane interface (∼0.1 g/L) was more than an order of magnitude lower than in DI water–cyclohexane (∼1.94 g/L), reflecting the strong influence of dissolved ions on surfactant adsorption. Interfacial dilatational measurements showed a clear maximum in the complex modulus |E*| (∼32 mN/m at 0.01 g/L SDS in PW), more than twice the maximum observed for DI water. The phase angle also increased rapidly with SDS concentration, indicating an earlier transition toward viscous-dominated interfacial behavior in PW. Time resolved droplet size distributions for oil-in-PW emulsions demonstrated that increasing SDS concentration produced smaller initial droplets and progressively reduced droplet growth over 24 h, consistent with enhanced reduction of coalescence at higher surfactant levels. Together, these results show that PW chemistry fundamentally modifies surfactant CMC, interfacial rheology, and droplet-scale emulsion behavior, providing fundamental insight into how water composition influences surfactant-based interfacial behavior relevant to oil–water separation and PW treatment.

## Graphical abstract

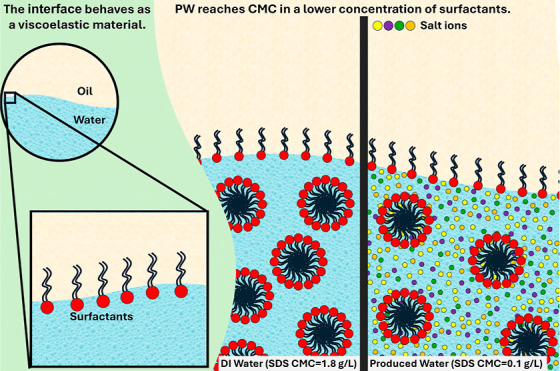

## 1 Introduction

Produced water (PW) is the largest waste stream from oil production, with over 900 billion gallons produced annually in the US alone originating from formation water, injected water for enhanced oil recovery, and aquifer breakthrough. It contains hydrocarbons, salts, surfactants, and other organic and inorganic species that strongly affect oil–water interfacial tension (IFT) and viscoelastic behavior.^1–7^ In production and treatment facilities, turbulence and pumping frequently generate oil-in-water emulsions that can be difficult to separate, and they also increase the operation costs and limit opportunities for reuse or downstream treatment. This has further motivated extensive research into PW treatment and reuse.^8^ Understanding the interfacial behavior and its mechanical characteristics is important for optimizing separation processes and resource recovery in the oil and gas industry.^9,10^

In oil production, water is often injected into wells to increase recovery rates, which can lead to the mixing of oil and water. Crude oil inherently contains natural surfactants, such as asphaltenes, resins, and polymers. The turbulent flow in these multiphase systems can result in the formation of stable oil–water emulsions, facilitated by these surfactants’ emulsifying properties.^11^ Without suitable stabilizers, emulsions are thermodynamically unstable systems that tend to separate back into the oil and water phases over time due to several instability mechanisms, including flocculation, gravitational separation, and coalescence.^12^

IFT is widely used to describe oil–water interactions and to evaluate the effect of surfactants and additives but equilibrium IFT alone often fails to predict emulsion stability because droplet coalescence and film drainage also depend on the mechanical response of the interfacial film to deformation.^13–15^ Interfacial dilatational rheology quantifies this response through the elastic (storage) modulus (*E*′), viscous (loss) modulus (*E*″), complex modulus (E*), and phase angle, which together describe how energy is stored and dissipated during cyclic area changes. These viscoelastic properties influence droplet collision efficiency, film rupture, and ultimately the evolution of emulsion droplet size distributions over time.

Surfactants actively modify IFT and interfacial rheology and are therefore central to emulsion control in petroleum systems.^16,17^ Sodium dodecyl sulfate (SDS), a well-characterized anionic surfactant, is commonly used as a model surfactant in interfacial studies due to its well-defined adsorption behavior and its strong ability to reduce IFT and influence emulsion stability. Cyclohexane is selected here as a representative nonpolar hydrocarbon with well-documented interfacial properties, enabling systematic evaluation of how SDS and aqueous phase composition couple to interfacial mechanics and emulsion behavior.^11,13,18^ Although real PW contains more complex mixtures of hydrocarbons, natural surfactants such as asphaltenes and resins, and other organic compounds that can influence interfacial behavior, simplified model systems are commonly used to isolate and better understand individual controlling factors. The synthetic PW used in this study reproduces the ionic composition that strongly affects interfacial behavior in field applications; however, real PW emulsions may show additional complexity because of their chemically diverse components.

Previous studies have successfully applied interfacial dilatational rheology to simplified oil–water and oil–brine systems using well-defined surfactants under controlled conditions.^2,19–21^ However, these model systems do not capture the chemical complexity of realistic PW, where high salinity, multivalent ions, and surfactant ion interactions can substantially alter interfacial structure and dynamics.^13,17,21–27^ A recent comprehensive review by the authors summarized current knowledge on the interfacial properties of PW and highlighted key knowledge gaps in understanding PW–oil interfacial phenomena.^2^ Although the bulk composition of PW has been extensively characterized,^28,29^ quantitative measurements of dilatational interfacial viscoelastic moduli (*E*′, *E*″, and E*) at oil–PW interfaces remain limited. Consequently, the connection between interfacial viscoelastic properties and droplet size evolution in PW emulsions has not yet been systematically investigated.

This work addresses this gap by combining dynamic interfacial measurements with direct emulsion characterization for synthetic PW–cyclohexane systems containing SDS. Using the pendant drop method under oscillatory conditions, the study quantifies IFT and dilatational moduli for DI water–cyclohexane and PW–cyclohexane interfaces as functions of SDS concentration and oscillation frequency. In parallel, oil-in-PW emulsions are prepared at selected SDS concentrations, and droplet size distributions are monitored over time to assess emulsion stability under conditions relevant to petroleum production. Together, these measurements provide a coherent picture linking PW chemistry, surfactant dosage, interfacial rheology, and the temporal evolution of droplet size in oil-in-PW emulsions, providing fundamental insights relevant to separation processes in complex PW environments.

## 2 Materials and Methods

### 2.1 Materials and Fluid Preparation

The solutions used in this study included deionized (DI) water having a resistivity of 18.2 MΩ cm, cyclohexane (≥98% purity, Sigma-Aldrich) as the organic phase, and sodium dodecyl sulfate (SDS, ≥98% purity, Sigma-Aldrich) as the anionic surfactant. Additionally, synthetic PW was prepared based on the composition reported by ref 30 consisting of sodium chloride (NaCl), magnesium sulfate heptahydrate (MgSO_4_·7H_2_O), calcium chloride dihydrate (CaCl_2_·2H_2_O), sodium bicarbonate (NaHCO_3_), sodium bromide (NaBr), potassium chloride (KCl), and iron­(II) sulfate heptahydrate (FeSO_4_·7H_2_O) (Fisher Scientific); strontium chloride hexahydrate (SrCl_2_·6H_2_O) and sodium metasilicate (Na_2_SiO_3_) (Sigma-Aldrich, St. Louis, Missouri, USA); and sodium sulfate (Na_2_SO_4_) (J.T. Baker) as shown in Table 1. All materials were used as received without further purification.

**Table 1:** Recipe Used for Synthetic PW^30^

chemical	formula	concentration (mg/L)
sodium chloride	NaCl	5020.90
magnesium sulfate heptahydrate	MgSO_4_·7H_2_O	3361.02
calcium chloride dihydrate	CaCl_2_·2H_2_O	4641.78
sodium bicarbonate	NaHCO_3_	407.80
sodium bromide	NaBr	24.47
strontium chloride hexahydrate	SrCl_2_·6H_2_O	81.19
potassium chloride	KCl	139.19
iron(II) sulfate heptahydrate	FeSO_4_·7H_2_O	10.00
sodium sulfate	Na_2_SO_4_	539.56
sodium silicate	Na_2_SiO_3_	139.08

### 2.2 Pendant-Drop Tensiometry and Interfacial Rheology

IFT and interfacial dilatational rheology measurements were performed using a pendant-drop tensiometer (ramé-hart Instrument Co.) equipped with an automated dispensing system (p/n 100-22), an oscillation module (p/n 100-28), and a high-speed camera. Image acquisition and analysis were conducted using DROPimage software (ramé-hart Instrument Co.). A schematic of the experimental setup is shown in Figure 1.

**Figure 1:**
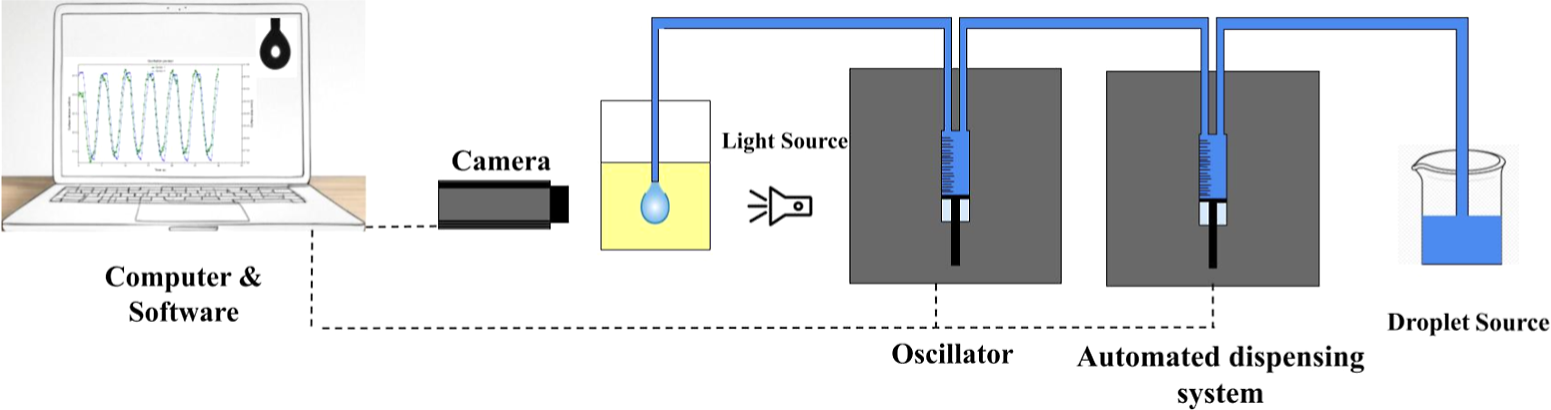
Experimental setup for dynamic IFT and viscoelastic property measurements.

In the pendant-drop configuration, a droplet suspended from a capillary needle adopts a shape governed by the balance between gravitational and interfacial forces.^31^ The shape of a pendant drop is governed by the Bond number (Bo), a dimensionless quantity that represents the ratio of gravitational to interfacial forces.^32^

1 $$Bo\equiv \frac{\Delta \rho gR_{0}^{2}}{\gamma }$$

where γ is the IFT; Δρ = ρ_d_ – ρ is the density difference between droplet density ρ_d_ and continuous phase density ρ; *g* is the gravitational acceleration; and *R*_0_ is the radius of the apex of the drop.

To probe interfacial mechanical behavior, dilatational rheology measurements were performed by applying controlled sinusoidal oscillations to the droplet area while monitoring the resulting IFT response.^33^ The resistance of the interface to area changes is quantified by the complex interfacial dilatational modulus E*, defined as

2 $$E^{*}=\frac{d\gamma }{d\ln A}=E^{\prime }+iE^{\prime \prime }$$

where *E*′ is the elastic (storage) modulus and *E*″ is the viscous (loss) modulus. The magnitude of the complex modulus and its components are given by

3 $$\left|E^{*}\right|=E\prime^{2}+E{\prime \prime }^{2}$$

4 $$E^{\prime }=\left|E^{*}\right|\cos\delta$$

5 $$E^{\prime \prime }=\left|E^{*}\right|\sin\delta$$

6 $$\tan\left(\delta \right)=E^{\prime \prime }/E^{\prime }$$

where δ is the phase angle between the applied interfacial area oscillation and the IFT response. A low phase angle indicates predominantly elastic behavior, whereas higher phase angles reflect increased viscous dissipation. These parameters provide quantitative insight into interfacial film strength, energy dissipation, and resistance to deformation under dynamic conditions relevant to droplet collisions and emulsion stability. Figure 2 schematically illustrates the phase lag between interfacial area strain and IFT during oscillatory tests.

**Figure 2:**
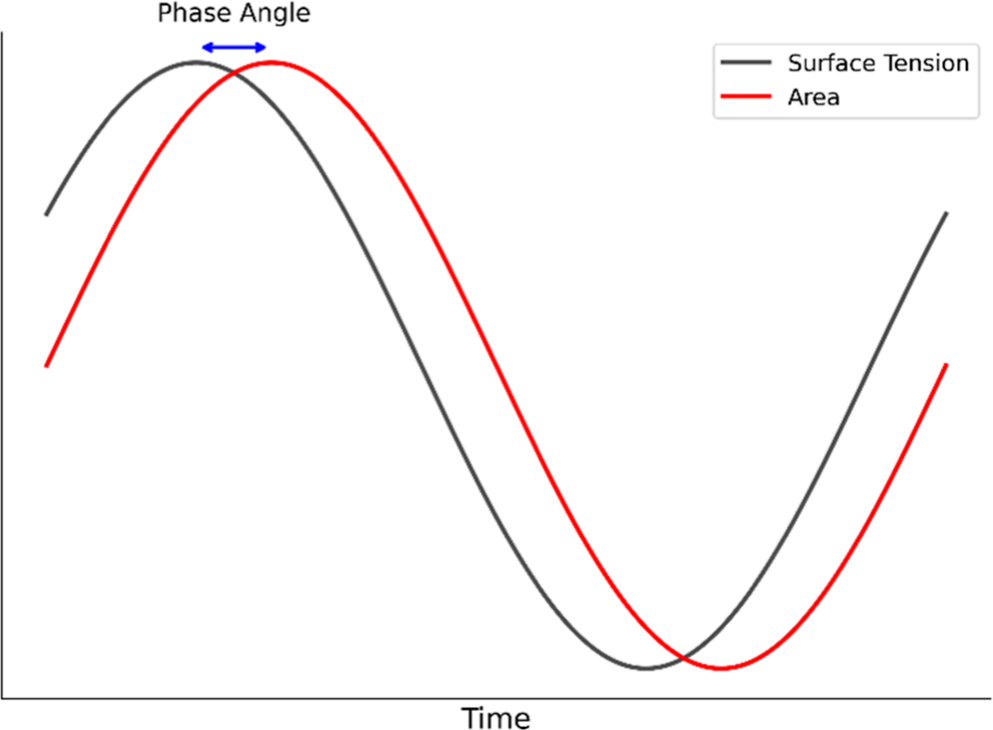
Oscillatory interfacial dynamics showing the phase angle (δ) between area (A) strain and IFT (γ) response.

### 2.3 Experimental Conditions and Measurement Protocol

All tests were conducted at ambient laboratory conditions, with a temperature of approximately 23 ± 1 °C and relative humidity around 50–55%. Droplets were allowed to equilibrate for 1000 s before data acquisition. IFT was recorded at a sampling rate of 1 Hz during the equilibrium period. Unless otherwise stated, at least three independent droplets were analyzed for each condition, and results are reported as mean ± standard deviation. Straight needles with an outer diameter (OD) of 1 mm were used for water–air, cyclohexane–air, and PW droplet-in-cyclohexane measurements, while an inverted needle (OD = 0.711 mm) was used for cyclohexane droplets formed in aqueous phases. Bond numbers were calculated using eq 1 for all systems; the maximum Bond number was 0.34, indicating sufficient droplet deformation for reliable pendant-drop shape analysis.^34^

Baseline measurements were first performed for water–air, cyclohexane–air, and water–cyclohexane systems to validate the tensiometer by comparison with literature values. Next, the effect of SDS on water–cyclohexane interfacial properties was examined. For IFT and CMC determination, SDS concentrations between 0.1 and 2.5 g/L were prepared in DI water as the continuous phase. For dilatational rheology, SDS concentrations of 0.001, 0.01, 0.1, and 1 g/L were investigated. The linear viscoelastic strain range was first determined by varying the droplet area deformation from 2 to 10% at a fixed oscillation period. After confirming the linear viscoelastic region, area oscillations of 5% around the initial droplet area were applied at frequencies from 0.1 to 0.5 Hz. For each frequency, six oscillation cycles were recorded with 200 data points per cycle. The same protocol was then applied to PW–cyclohexane systems. Synthetic PW (with or without SDS) served as the droplet phase in cyclohexane. SDS concentrations of 0.001, 0.01, 0.1, and 1 g/L in PW were studied for both IFT and dilatational rheology. At least three independent droplets were analyzed for each fluid composition and frequency. Reported values of IFT and moduli correspond to the mean of these replicates, and error bars represent one standard deviation.

### 2.4 Emulsion Preparation and Droplet Size Analysis

Oil-in-PW emulsions were prepared to directly evaluate the effects of SDS concentration on droplet size distribution. Synthetic PW containing SDS at selected concentrations (0.001, 0.01, 0.1, and 1 g/L) was used as the continuous phase, while cyclohexane served as the dispersed oil phase at an oil volume fraction of 1%. Emulsification was carried out using an Ultra-Turrax digital homogenizer operated at 10,000 rpm to ensure consistent energy input across all experiments. Prior to emulsification, the required amount of SDS was added to the continuous phase and preshared at 10,000 rpm for 5 min to ensure complete surfactant dispersion. The oil phase was then added dropwise to the surfactant containing continuous phase while the homogenizer was running.^12^ Following completion of oil addition, homogenization was continued for an additional 5 min to ensure uniform droplet formation. Samples were withdrawn at predetermined aging times (0 min, 10 min, 60 min, 12 h, and 24 h) for analysis. Time-resolved imaging approaches can be useful for monitoring dynamic soft-material and multiphase systems under transient conditions.^35^ Droplet size distributions were measured using an Olympus BX53 cross-polarized optical microscope, which is equipped with a high-speed camera. For microscopy, at least five images at different locations were captured for each sample, and droplet diameters were extracted using the Fiji ImageJ software. Number-based droplet size distributions were constructed, and mean diameters and standard deviations were reported for each condition and time.

To further evaluate surfactant availability during emulsification, the ratio of the total surfactant to the estimated total oil–water interfacial area generated at the initial time was approximated for each emulsion condition. The total interfacial area (TIA) was estimated assuming a spherical droplet:^36^

7 $$TIA=\frac{6V_{oil}}{d}$$

where TIA is the estimated total interfacial area, *V*_oil_ is the total dispersed oil volume, and *d* is the measured mean droplet diameter at the initial time. The surfactant to interfacial area ratio was then estimated by dividing the total SDS mass in the continuous phase by the calculated interfacial area.

## 3 Results and Discussion

### 3.1 Effect of SDS on IFT and CMC Behavior

Baseline surface and interfacial tension measurements were conducted for water–air, cyclohexane–air, and water–cyclohexane systems to verify the accuracy and reliability of the pendant-drop tensiometry setup (Table 2). The measured values agree with previously reported data within about 1% for water–air and cyclohexane–air and within 2% for water–cyclohexane, which confirms the accuracy of the pendant-drop setup and provides confidence in the subsequent measurements on SDS solutions and produced-water systems.

**Table 2:** Surface/Interfacial Tension of Water–Air, Cyclohexane–Air, and Water–Cyclohexane; Literature and Measured Values

	surface/interfacial tension (mN/m, at room temperature)	
phases	reported value in literature	measured value in this study
water–air	72.8 ^37^	72.3 ± 0.68
cyclohexane–air	24.5 ^38^	24.8 ± 0.20
cyclohexane–water	48.9 ^39^	47.9 ± 1.26

Figure 3 compares the effects of SDS concentration on water–air surface tension and cyclohexane–water IFT. Increasing SDS concentration reduced both surface and interfacial tension values toward a plateau region.

**Figure 3:**
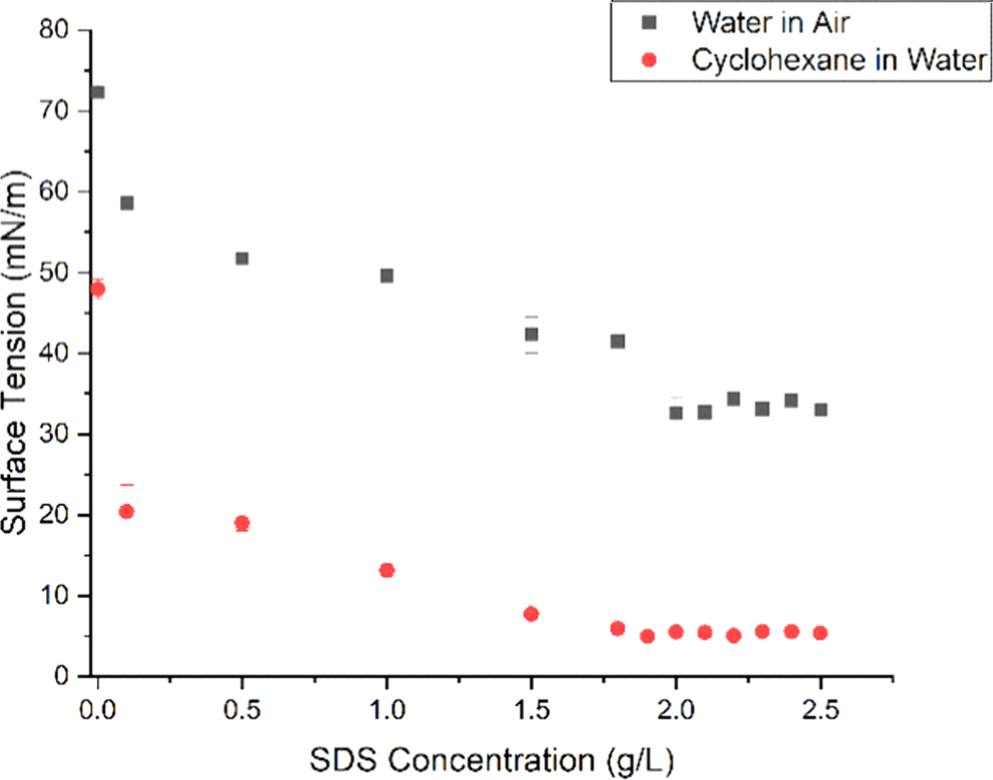
Cyclohexane–water IFT and water–air surface tension at different SDS concentrations.

To better resolve the transition region, the cyclohexane–water IFT data were replotted on a logarithmic SDS concentration scale (Figure 4). The IFT decreased from 47.9 ± 1.26 mN/m without SDS to 5.91 ± 0.03 mN/m at 1.8 g/L SDS, after which only minor changes were observed. The apparent CMC was estimated from the intersection between the linear fit to the decreasing region and the plateau region, giving an apparent CMC of 1.94 g/L. This value is approximately 0.43 g/L lower than the reported CMC in pure water (≈2.36 g/L at 25 °C), suggesting that the presence of the oil phase facilitates micellization at lower bulk surfactant concentrations. The corresponding water–air surface tension data in Figure 3 gave a CMC range of approximately 2–2.5 g/L, consistent with literature values.

**Figure 4:**
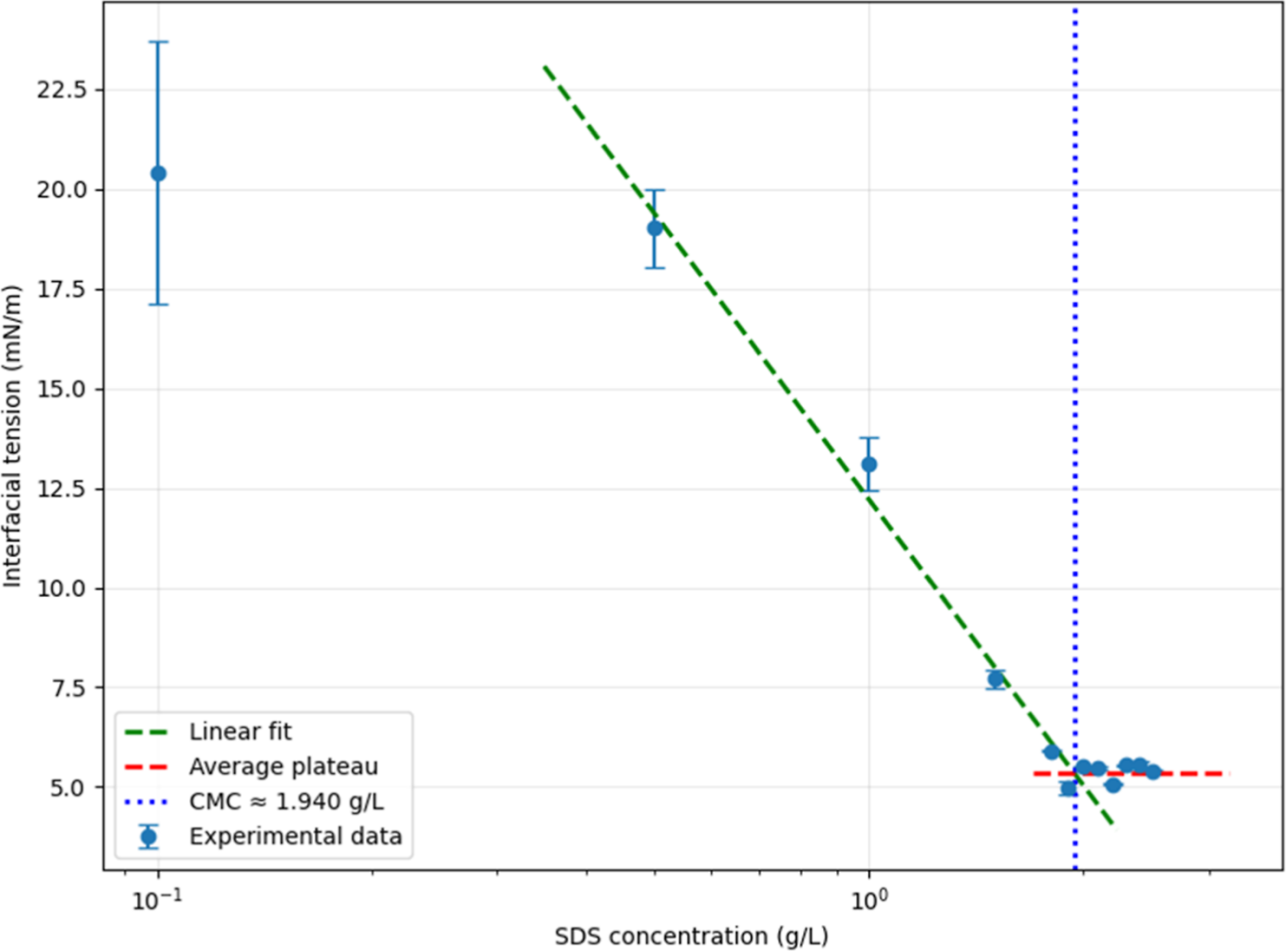
Determination of the apparent CMC for the cyclohexane–water system.

The interfacial behavior of synthesized PW in contact with cyclohexane was examined and compared with that of DI water to investigate the effect of dissolved PW components on surfactant performance. In the absence of a surfactant, the IFT between synthesized PW and cyclohexane was measured as 44.4 ± 0.32 mN/m, which is 4.5 mN/m lower than that of the DI water–cyclohexane system (47.9 ± 1.26 mN/m; Table 2). This reduction reflects the influence of dissolved salts and ionic species in PW on baseline interfacial properties.

Figure 5 shows the variation of IFT between synthesized PW and cyclohexane as a function of SDS concentration on a logarithmic scale. Compared to the DI water–cyclohexane system, the PW–cyclohexane interface exhibited a much stronger sensitivity to SDS addition. A sharp decrease in IFT was observed at low surfactant concentrations, followed by an early plateau. Above 0.1 g/L SDS, further increases in concentration produced little change in IFT, indicating saturation of the interface. The apparent CMC was estimated from the intersection between the linear fit and plateau region, giving an apparent CMC of 0.1 g/L SDS. This value is more than an order of magnitude lower than the CMC determined for the DI water–cyclohexane system (≈1.94 g/L).

**Figure 5:**
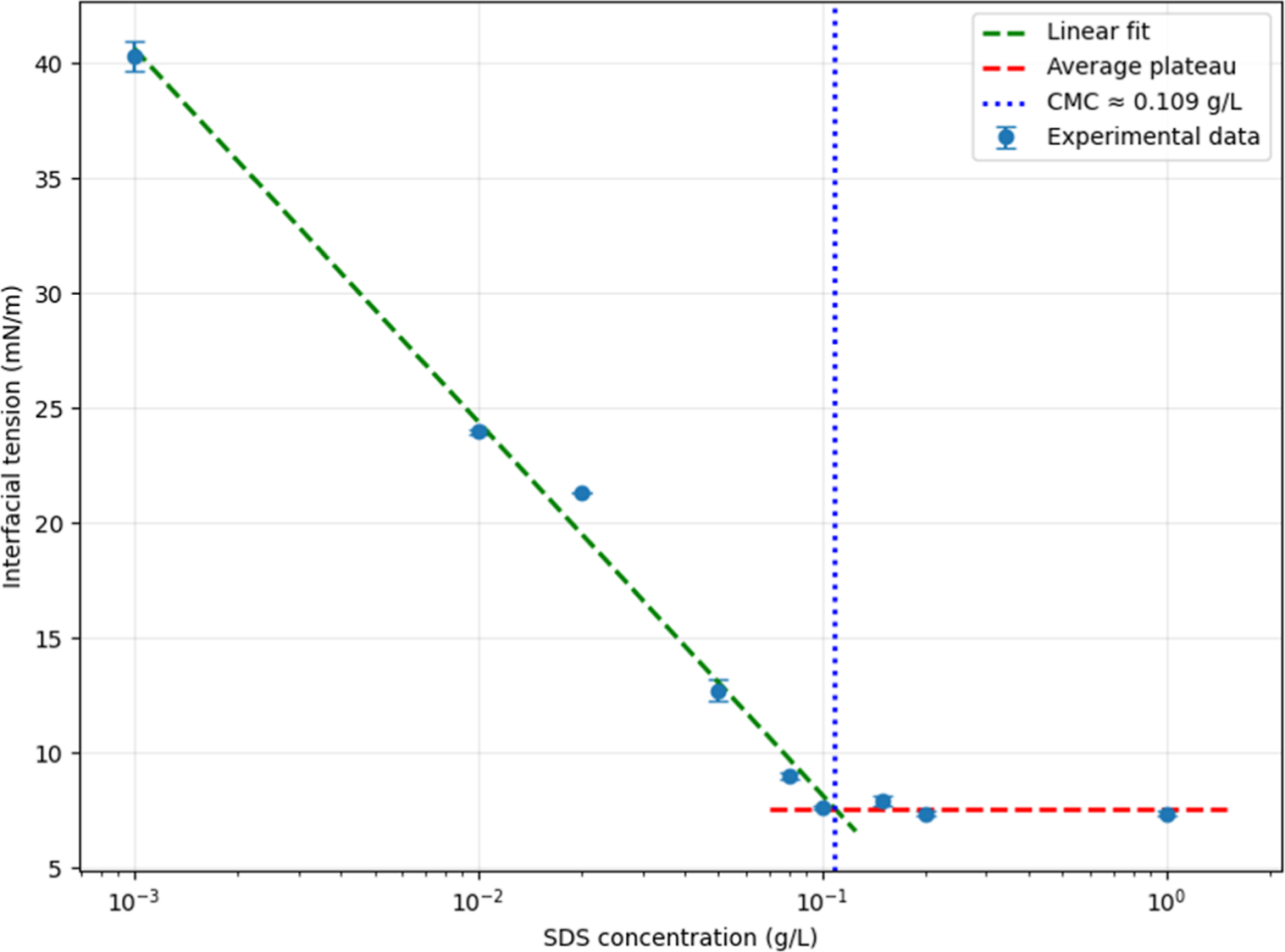
Determination of the apparent CMC for the PW–cyclohexane system.

The lower CMC in the PW system can be attributed to the high ionic strength and presence of multivalent ions, which screen electrostatic repulsion between the anionic SDS headgroups and reduce surfactant solubility in the aqueous phase.^40^ These effects promote earlier interfacial saturation and micelle formation at substantially lower surfactant concentrations. While enhanced surfactant efficiency at low concentrations is advantageous for reducing IFT, these ionic effects are also expected to alter interfacial transport and viscoelastic response, motivating the dilatational rheology measurements discussed in the following section.

In addition to the overall ionic strength, specific ion effects likely contribute to the observed behaviors. Previous studies have shown that multivalent ions such as Ca^2+^ and Mg^2+^ can reduce the CMC of anionic surfactants more effectively than monovalent salts by strongly screening electrostatic repulsion between surfactant headgroups and altering adsorption and micellization behavior.^40,41^ Molecular thermodynamic modeling studies have also demonstrated pronounced ion-specific effects on SDS micellization and interfacial electrostatics in mono- and divalent salt solutions.^42,43^ Therefore, the reduction in CMC and altered interfacial behavior observed in PW are likely influenced by both the high total ionic strength and ion-specific interactions associated with multivalent ions present in the synthetic PW composition.

### 3.2 Interfacial Dilatational Rheology of DI Water–Cyclohexane Interfaces

Dilatational rheology was measured for cyclohexane droplets in aqueous SDS solutions at 0.001, 0.01, 0.1, and 1 g/L over frequencies from 0.1 to 0.5 Hz. Dilatational moduli for all SDS concentrations and frequencies are provided in Tables S1–S4 (Supporting Information). At the lowest SDS concentration (0.001 g/L), the interface is strongly elastic: *E*′ is about 6–7 mN/m across the frequency range, whereas *E*″ and the phase angle remain small (≈5°), indicating limited viscous dissipation. Increasing SDS to 0.01 g/L raises both *E*′ and *E*″, and the phase angle grows to about 11–14°, consistent with the formation of a more viscoelastic interfacial layer. A further increase to 0.1 g/L produces the largest moduli, with *E*′ and *E*″ both reaching their maxima and phase angles in the range of 22–26° over all frequencies. This indicates that near 0.1 g/L, the interface forms a dense surfactant layer that both stores and dissipates interfacial energy efficiently. At 1 g/L SDS, the storage modulus decreases relative to 0.1 g/L, whereas the loss modulus and phase angles remain comparatively high, especially at low frequencies. The similar phase angles at 0.1 and 1 g/L suggest that once the interface is saturated, additional surfactant primarily affects viscous dissipation rather than increasing interfacial elasticity.

Figure 6 summarizes the dependence of storage (*E*′), loss (*E*″), and complex modulus (|E*|) on SDS concentration and oscillation frequency. A clear frequency dependence is observed in both the magnitude of the moduli and the SDS concentration at which the maximum response occurs. At the lowest frequency (0.1 Hz), the complex modulus reaches its maximum at 0.01 g/L SDS, indicating that under slow interfacial deformation, the surfactant layer has sufficient time to relax and redistribute, leading to a mechanically optimal interface at relatively low surface coverage.

**Figure 6:**
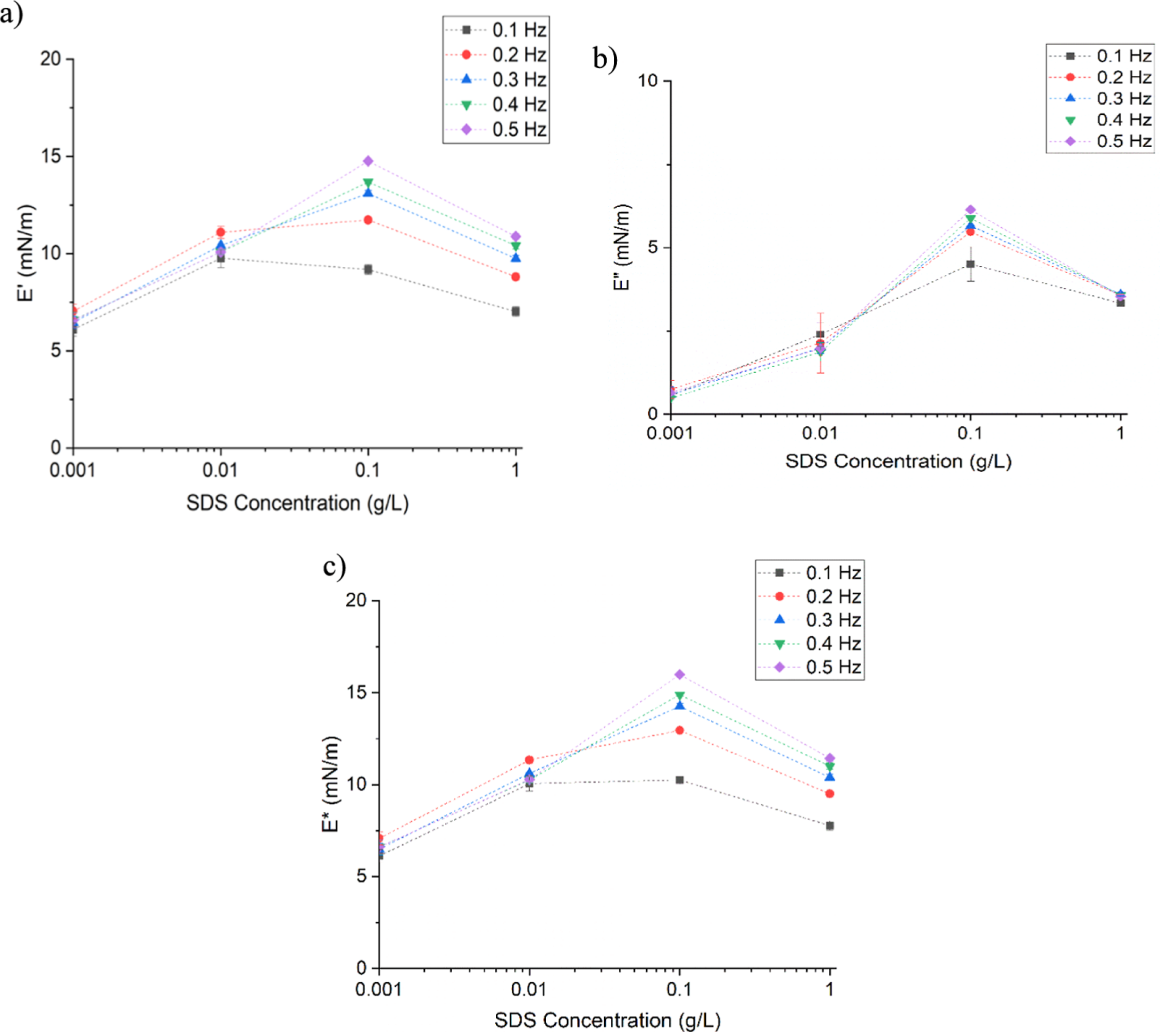
Measured moduli for cyclohexane (droplet phase) in DI water + SDS (external phase): (a) storage modulus (*E*′), (b) loss modulus (*E*″), and (c) complex modulus (E*).

In contrast, at higher frequencies (0.2–0.5 Hz), the maximum in |E*| consistently shifts to 0.1 g/L SDS. This shift may reflect the reduced ability of surfactant molecules to reorganize during faster interfacial oscillations, suggesting that a higher interfacial surfactant density is required to maintain interfacial rigidity and viscoelastic resistance. For all frequencies, increasing SDS concentration beyond 0.1 g/L does not further increase |E*|, and in some cases leads to a reduction in *E*′, suggesting that interfacial saturation primarily enhances viscous dissipation rather than elastic energy storage. These results suggest that the interfacial rheological response is governed by a competition between surfactant relaxation times and the imposed deformation frequency, resulting in a frequency-dependent optimum in interfacial viscoelasticity. This dynamic coupling may influence droplet coalescence behavior and emulsion stability, as discussed later using droplet size distribution measurements.

### 3.3 Interfacial Dilatational Rheology of the PW–Cyclohexane Interface

In the absence of a surfactant, the IFT between synthetic PW and cyclohexane was 44.4 ± 0.32 mN/m, about 4.5 mN/m lower than for DI water–cyclohexane, reflecting the influence of dissolved salts and ionic species on baseline interfacial properties. With increasing SDS concentration, the PW–cyclohexane IFT decreased sharply and showed minimal additional reduction beyond approximately 0.1 g/L SDS, suggesting the onset of interfacial saturation and an apparent CMC substantially lower than that of the DI water–cyclohexane system. This behavior is consistent with “specific ion effects”, where high ionic strength and multivalent ions (e.g., Ca^2+^, Mg^2+^, SO_4_^2–^) screen electrostatic repulsion between SDS headgroups, promoting earlier interfacial saturation and micellization.^44,45^

Dilatational rheology was measured for cyclohexane in PW containing 0.001, 0.01, 0.1, and 1 g/L SDS over 0.1–0.5 Hz. Complete numerical data are provided in Tables S5–S8 (Supporting Information). As in DI water, increasing SDS in PW reduced the gap between storage modulus *E*′ and loss modulus *E*″, but the evolution of phase angle was more pronounced.

Figure 7 summarizes the phase angle ranges for PW + SDS and DI water + SDS, showing that PW exhibits a much faster increase in the phase angle with SDS concentration. This indicates that the PW interface becomes more viscous than the DI water interface at comparable SDS levels, consistent with enhanced damping and slower interfacial rearrangements in the presence of ions.

**Figure 7:**
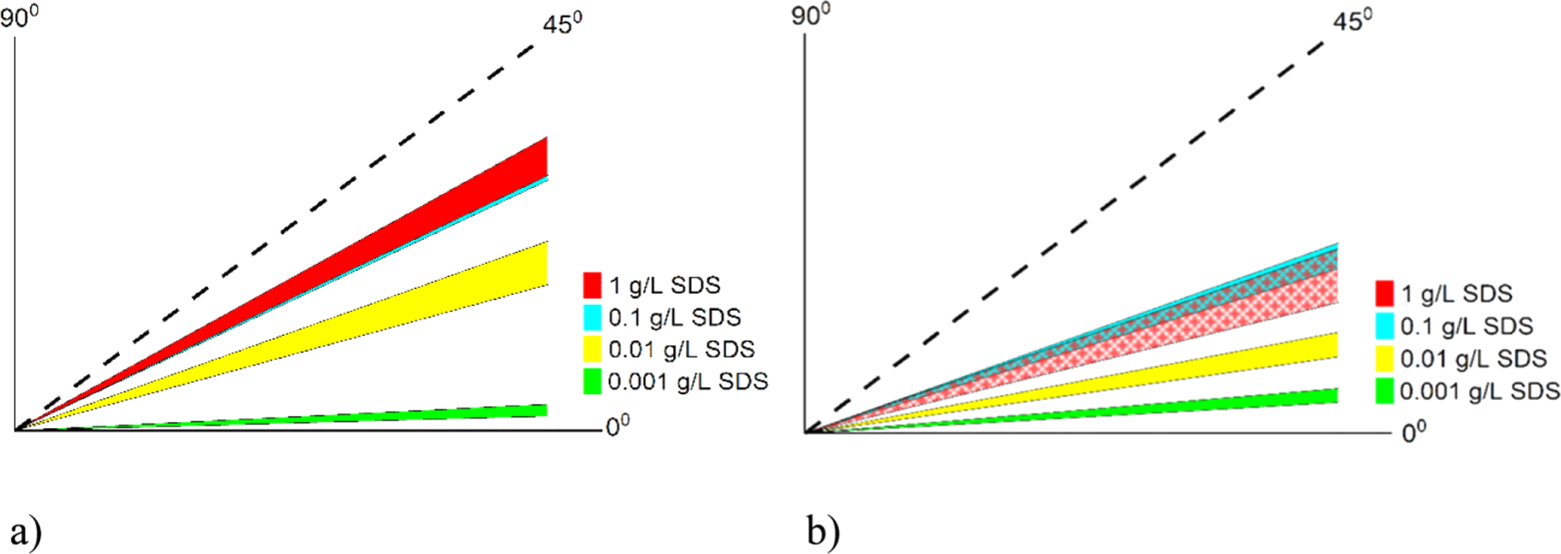
Variation in the phase angle for (a) PW + SDS and (b) DI water + SDS in contact with cyclohexane; each region represents the range of minimum and maximum phase angle values recorded for the solution at frequencies between 0.1 and 0.5 Hz.

Figure 8 presents the frequency dependence of *E*′, *E*″, and |E*| for PW + SDS. Both moduli increase markedly when SDS rises from 0.001 to 0.01 g/L, and the complex modulus reaches its maximum at 0.01 g/L across all frequencies (up to about 32 mN/m at 0.5 Hz). This concentration corresponds to the formation of mechanically strong viscoelastic film in which both elastic and viscous contributions are significant. Above 0.01 g/L, both *E*′ and *E*″ decrease, despite the lower equilibrium IFT, suggesting that interfacial saturation and reduced lateral mobility of SDS limit dynamic rearrangements and weaken the dilatational response. Compared to the DI water system, the PW interface exhibits higher phase angles, indicating a larger viscous contribution to the interfacial response in the presence of dissolved ions. These results demonstrate that PW composition and surfactant dosage jointly influence not only equilibrium IFT and CMC behavior but also the dynamic interfacial mechanics governing emulsion stability, as explored further through droplet-size evolution measurements in the following section.

**Figure 8:**
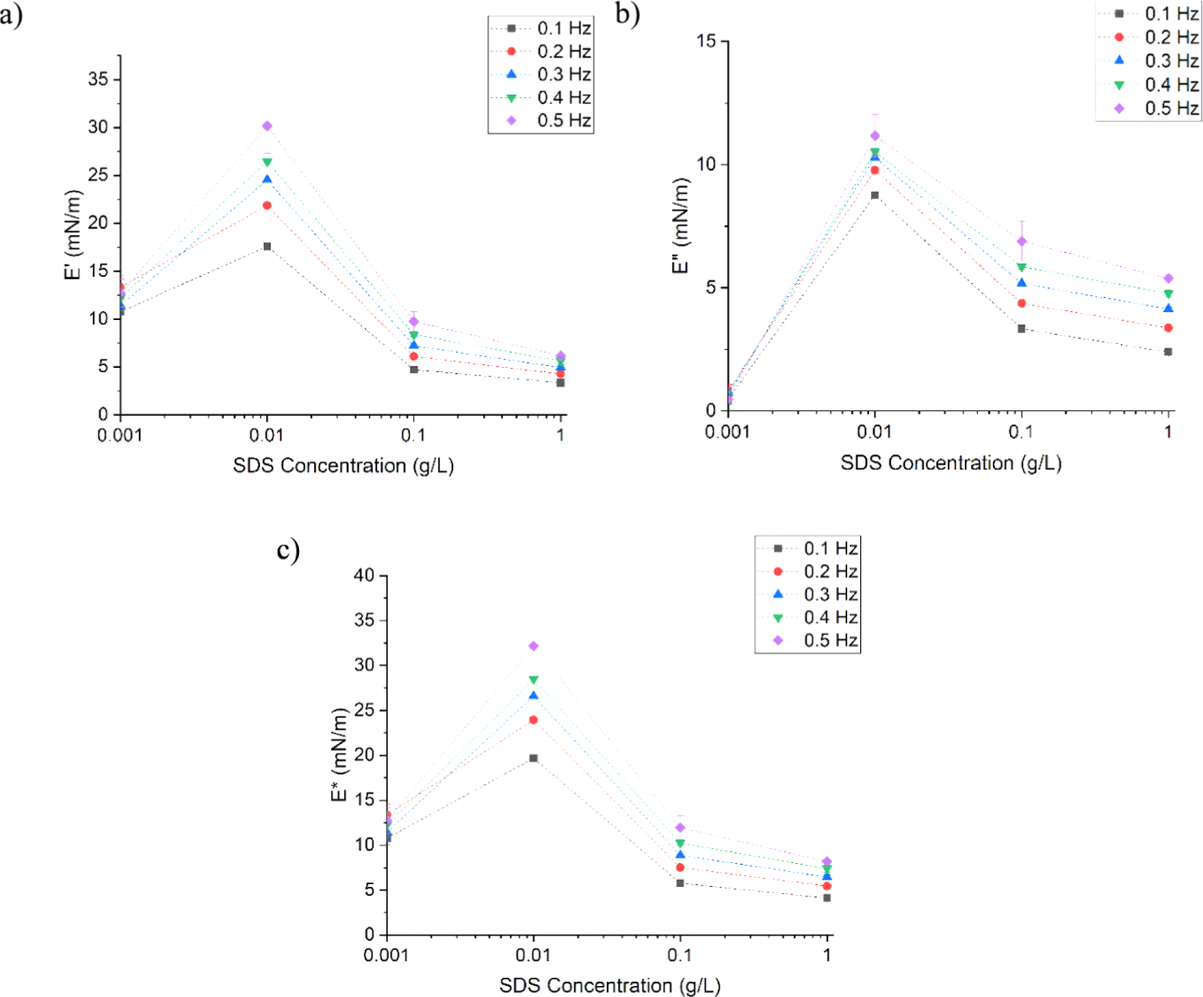
Measured moduli for PW + SDS (droplet phase) in cyclohexane (external phase): (a) storage modulus (*E*′), (b) loss modulus (*E*″), and (c) complex modulus (E*).

### 3.4 Droplet-Size Distribution Evolution in Oil-in-PW Emulsions

Droplet-size distributions were analyzed to evaluate how the SDS concentration influences the temporal evolution of dispersed oil droplets in synthetic PW emulsions. Representative optical micrographs of emulsions prepared with different SDS concentrations shortly after emulsification are shown in Figure 9, illustrating the initial droplet morphology prior to the quantitative droplet size analysis.

**Figure 9:**
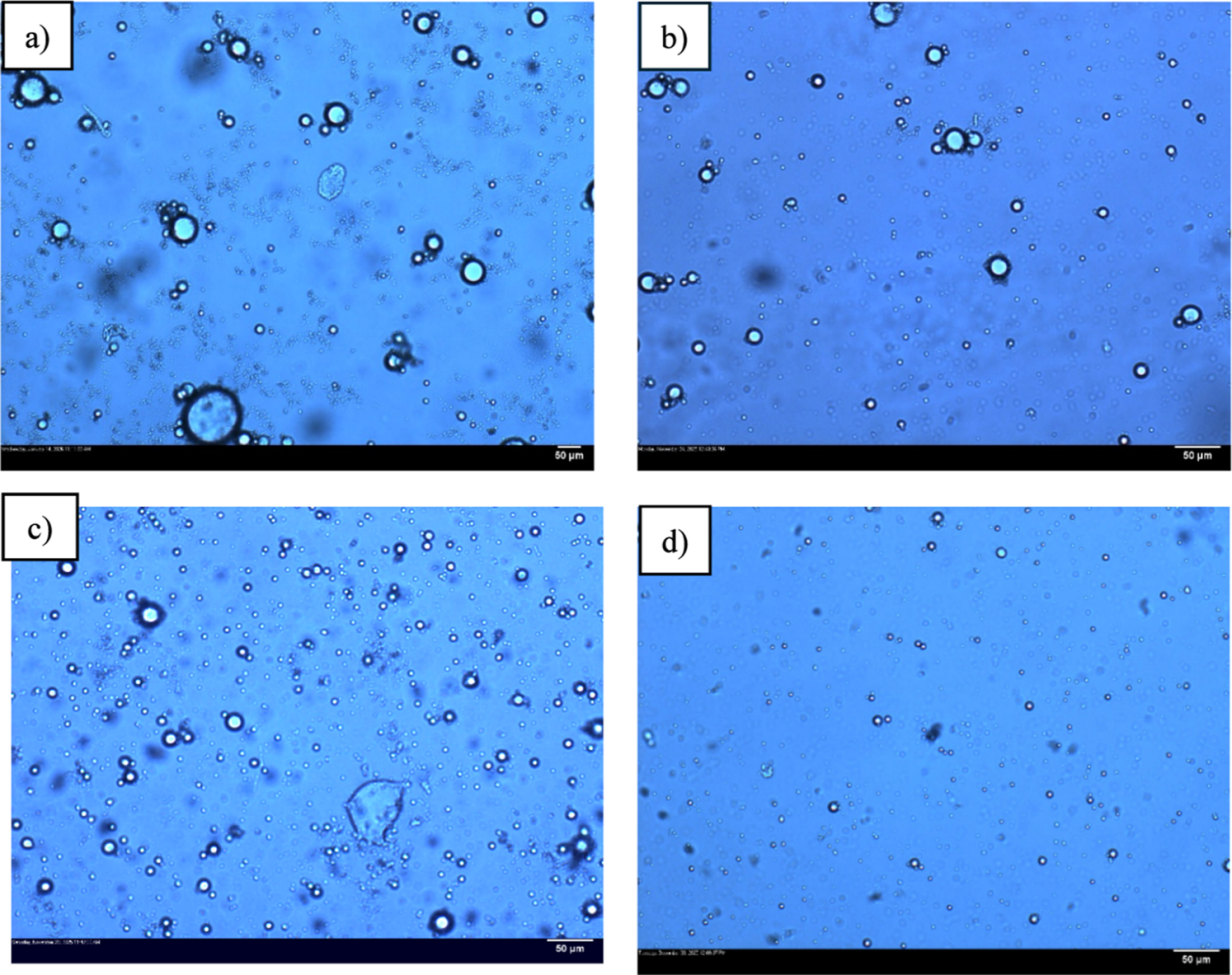
Representative optical micrographs of oil-in-produced-water emulsions at the initial time after emulsification with different SDS concentrations: (a) 0.001 g/L, (b) 0.01 g/L, (c) 0.1 g/L, and (d) 1 g/L SDS (scale bar = 50 μm).

Emulsions containing 1 vol % cyclohexane and varying SDS concentrations (0.001, 0.01, 0.1, and 1 g/L) were prepared under identical mixing conditions, and samples were collected at 0, 10, and 60 min, 12 h, and 24 h for image-based analysis. The corresponding mean droplet diameters are summarized in Table 3. Representative droplet size distributions for the lowest (0.001 g/L) and highest (1 g/L) SDS concentrations are shown in Figures 10 and 11, while the complete set of histograms is provided in the Supporting Information (Figures S1–S4).

**Table 3:** Time-Dependent Mean Droplet Diameters for Oil-in-PW Emulsions at Different SDS Concentrations

	mean diameter 0.001 g/L SDS (μm)	mean diameter 0.01 g/L SDS (μm)	mean diameter 0.1 g/L SDS (μm)	mean diameter 1 g/L SDS (μm)
0 min	16.6 ± 9.04	12.5 ± 5.45	10.5 ± 2.79	7.06 ± 2.14
10 min	25.7 ± 17.1	13.3 ± 5.47	10.7 ± 2.94	7.99 ± 2.75
60 min	30.4 ± 17.1	14.6 ± 6.20	12.7 ± 3.46	8.14 ± 2.53
12 h	31.77 ± 17.4	15.1 ± 4.80	13.09 ± 3.42	9.14 ± 2.30
24 h	42.1 ± 21.3	19.3 ± 6.35	14.1 ± 4.14	11.9 ± 4.42

**Figure 10:**
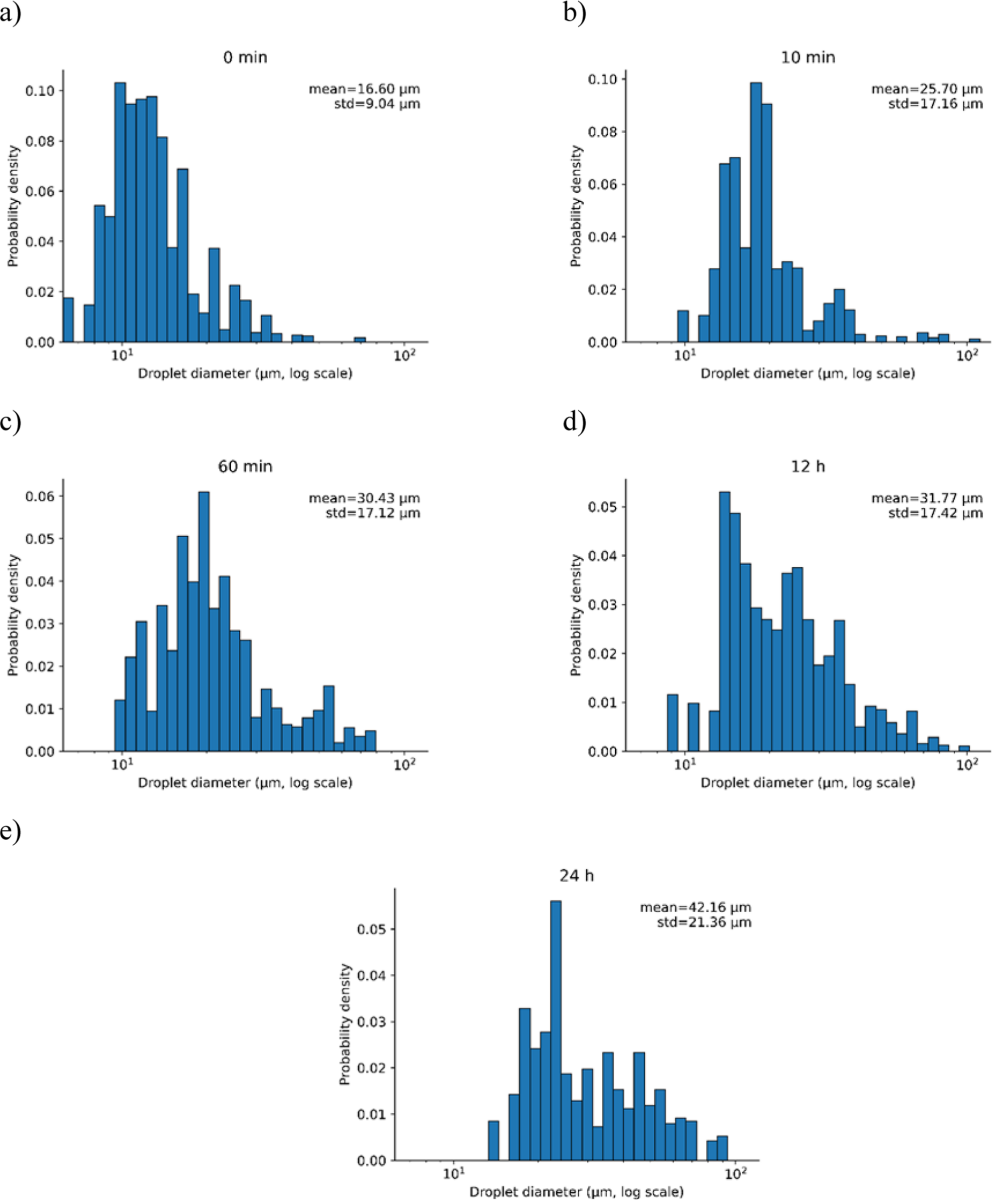
Probability density histograms of droplet diameter distributions for oil-in-PW emulsions containing 1 vol % cyclohexane in 99 vol % PW with 0.001 g/L SDS, measured at (a) 0 min, (b) 10 min, (c) 60 min, (d) 12 h, and (e) 24 h.

**Figure 11:**
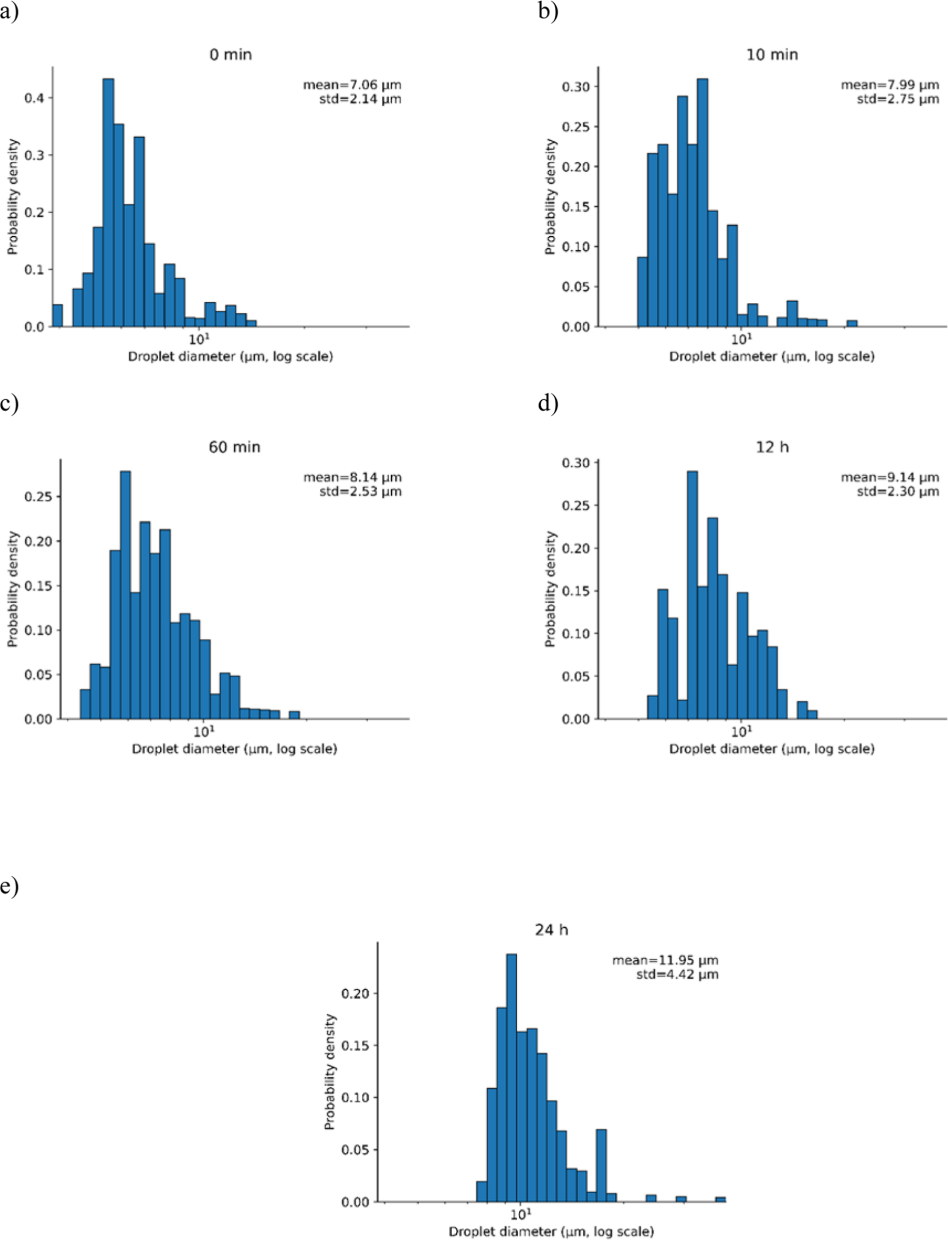
Probability density histograms of droplet diameter distributions for oil-in-PW emulsions containing 1 vol % cyclohexane in 99 vol % PW with 1 g/L SDS, measured at (a) 0 min, (b) 10 min, (c) 60 min, (d) 12 h, and (e) 24 h.

For emulsions containing 0.001 g/L SDS, significant droplet growth is observed during aging, with the mean droplet diameter increasing from 16.6 μm at the initial time to 42.1 μm after 24 h. This behavior suggests that the interfacial film formed at very low surfactant concentrations provides insufficient coverage of the oil–water interface (see the flowing section), resulting in limited resistance to droplet interactions. Increasing the SDS concentration to 0.01 g/L results in noticeably reduced droplet growth. Although the droplet size distributions still shift toward larger diameters with time, the changes are much smaller than those observed at 0.001 g/L. The mean droplet diameter increases from 12.5 μm initially to 19.3 μm after 24 h, and the distributions remain relatively narrow throughout the aging period. At 0.1 and 1 g/L SDS, only small changes in the droplet size distributions are observed over 24 h. Mean droplet diameters increase from 10.5 to 14.1 μm for 0.1 g/L SDS and from 7.06 to 11.9 μm for 1 g/L SDS, demonstrating a progressive suppression of droplet growth with increasing surfactant concentration. This consistent trend is in agreement with the IFT and dilatational rheology measurements, which showed that higher SDS concentrations reduce IFT and promote more persistent surfactant coverage of the PW–oil interface.

#### 3.4.1 Surfactant-to-Interfacial-Area Ratio Analysis

To further examine the relationship between surfactant availability and droplet size evolution, the ratio of total SDS mass to the estimated total oil–water interfacial area generated immediately after emulsification was calculated for each SDS concentration using the method described in Section 2.4. The calculated ratios are summarized in Table 4.

**Table 4:** Estimated Surfactant to Interfacial Area Ratios for Oil-in-PW Emulsions at Different SDS Concentrations Immediately after Emulsification

SDS (mg/L)	surface coverage ratio
0.001	0.27
0.01	2.00
0.1	16.8
1	113

The estimated surfactant-to-interfacial area ratio increased substantially with increasing SDS concentration. Although 0.01 g/L SDS produced the highest measured complex modulus, its lower surfactant-to-interfacial area ratio relative to 0.1 and 1 g/L SDS may suggest limited surfactant availability during emulsification, which could contribute to enhanced droplet coalescence and droplet growth over time.

## 4 Discussion

The droplet size growth in emulsions is closely linked to the mechanical properties of the interfacial film, which governs droplet deformation and coalescence during droplet–droplet interactions. In the PW–cyclohexane system studied here, the interfacial storage (*E*′), loss (*E*″), and complex (E*) moduli exhibited a strong dependence on SDS concentration, with a maximum in E* at 0.01 g/L SDS. At this concentration, the complex modulus reached 32 mN/m at 0.5 Hz, and the corresponding phase angle was relatively low, indicating an elastic interfacial response. These results suggest the formation of a mechanically strong interfacial film under small-amplitude oscillatory deformation.

Despite the high interfacial modulus observed at 0.01 g/L SDS, time-resolved droplet size measurements reveal that emulsions prepared at this concentration still experience considerable droplet growth over 24 h. Mean droplet diameters increase more rapidly at 0.01 g/L than at higher SDS concentrations, whereas emulsions containing 0.1 and 1 g/L of SDS consistently exhibit smaller mean droplet sizes and slower temporal growth.

This apparent discrepancy suggests that interfacial viscoelasticity measured on an equilibrated, single interface does not directly translate to population-level droplet behavior in emulsions. As discussed in Section 3.4.1, the estimated surfactant to interfacial area ratio at 0.01 g/L SDS remained substantially lower than at 0.1 and 1 g/L SDS, despite the higher measured complex modulus. This may indicate limited surfactant availability during emulsification, which could contribute to enhanced droplet coalescence and droplet growth over time.

At higher SDS concentrations (0.1 and 1 g/L), droplet size evolution was substantially suppressed even though the measured interfacial moduli decreased relative to their peak values. The substantially larger surfactant to interfacial area ratios at these concentrations likely promoted more persistent interfacial coverage during emulsification and droplet deformation. Although interfacial saturation reduces the dynamic dilatational elasticity reflected by lower *E*′ and higher phase angles, the fully covered surfactant layer may provide a persistent kinetic barrier to coalescence. Electrostatic repulsion between charged SDS headgroups and reduced IFT together limits film drainage and droplet merging, resulting in slower growth of the mean droplet diameter over time.

Overall, these results demonstrate that droplet size evolution in oil-in-PW emulsions is governed by a balance between the interfacial mechanical strength and surfactant availability. While a high interfacial modulus reflects the potential for a mechanically resistant film, effective suppression of droplet growth requires a sufficient amount of surfactant to ensure continuous and uniform interfacial coverage across the emulsion. The reduction in mean droplet size growth with increasing SDS concentration observed in this study underscores the importance of surfactant coverage and redistribution in determining emulsion microstructural evolution in ion-rich PW systems. Interfacial dilatational rheology therefore provides valuable mechanistic insights, but its implications for emulsion behavior must be interpreted in conjunction with bulk surfactant concentration and adsorption dynamics. Although the results suggest that surfactant availability and interfacial coverage play important roles in droplet size evolution, these effects were not directly measured in the present study. Future work incorporating adsorption kinetics, interfacial imaging, or direct interfacial coverage measurements would help to further verify the proposed mechanisms.

## 5 Conclusion

This study focused on elucidating the viscoelastic properties of PW emulsions by systematically investigating IFT, interfacial dilatational viscoelasticity, and droplet size evolution in synthetic PW in the presence of the anionic surfactant SDS. By combining pendant-drop tensiometry, oscillatory interfacial rheology, and time-resolved droplet-scale analysis, this work provides a comprehensive assessment of how ion-rich PW chemistry alters interfacial dynamics relative to idealized aqueous systems. The results show that PW chemistry strongly modifies surfactant performance at the oil–water interface. The IFT of the PW–cyclohexane system (44.4 ± 0.32 mN/m) was approximately 4.5 mN/m lower than that of the DI water–cyclohexane system. In addition, the apparent CMC decreased to ∼0.1 g/L SDS, compared with ∼1.94 g/L for DI water–cyclohexane and ∼2–2.5 g/L for water–air interfaces. Interfacial dilatational rheology revealed substantially higher interfacial stiffness in PW, with the complex modulus (E*) reaching ∼32 mN/m at 0.5 Hz, more than double that measured for DI water under comparable conditions. Droplet size measurements further demonstrated that increasing SDS concentration in PW leads to smaller initial droplets and progressively reduced droplet growth over time. For PW, mean droplet diameters increased from 16.4 to 42.1 μm at 0.001 g/L SDS over 24 h, while growth was strongly suppressed at higher concentrations, increasing only from 10.5 to 14.1 μm at 0.1 g/L and from 7.06 to 11.9 μm at 1 g/L. These results indicate that emulsion stability in oil-in-PW emulsions depends not only on interfacial viscoelasticity but also on surfactant availability relative to the generated interfacial area during emulsification. By explicitly linking PW chemistry, interfacial rheology, and droplet scale evolution, this study advances fundamental understanding of emulsion behavior in PW systems. These findings provide fundamental insight into interfacial phenomena relevant to PW treatment, emulsion management, and surfactant or demulsifier design in oilfield operations. Future work should extend this framework to real PW, alternative surfactant chemistries, and commercial demulsifiers to further optimize separation strategies under realistic operating conditions.

## Supplementary Material



## Notes

Dilatational rheological properties, including storage modulus (*E*′), loss modulus (*E*″), complex modulus (*E**), and phase angle, of cyclohexane–water interfaces containing 0.001, 0.01, 0.1, and 1 g/L SDS; dilatational rheological properties of cyclohexane–PW interfaces containing 0.001, 0.01, 0.1, and 1 g/L SDS; and probability density histograms of droplet size distributions for oil-in-PW emulsions containing 1 vol % cyclohexane and 99 vol % PW with SDS concentrations of 0.001, 0.01, 0.1, and 1 g/L measured during aging from 0 min to 24 h (PDF)
