# Supplementary material for: Viscoelastic Properties of Produced Water Emulsions
Source: Energy Fuels. 2026 Jun 23;40(26):14347–59. doi: 10.1021/acs.energyfuels.6c01394 (PMC13339165; doi:10.1021/acs.energyfuels.6c01394)
Supplement: Supplementary file 1 [file ef6c01394_si_001.pdf]

Supporting Information

Viscoelastic Properties of Produced Water Emulsions

Alireza Zahedi[1], Saeed Azizi[1], Mark Krzmarzick[2], Clint. P. Aichele[1*]

[1] School of Chemical Engineering, Oklahoma State University, Stillwater, OK, USA
[2] School of Civil and Environmental Engineering, Oklahoma State University, Stillwater, OK, USA

Table S1 Dilatational properties of cyclohexane in water +0.001 g/L SDS

**Frequency (Hz)**	**E' (mN/m)**	**E" (mN/m)**	**E* (mN/m)**	**Phase Angle (°)**
0.1	6.09 ± 0.34	0.56 ± 0.08	6.12 ± 0.33	5.31 ± 1.02
0.2	7.03 ± 0.38	0.75 ± 0.27	7.08 ± 0.36	6.15 ± 2.46
0.3	6.46 ± 0.32	0.59 ± 0.24	6.49 ± 0.34	5.14 ± 1.84
0.4	6.59 ± 0.39	0.48 ± 0.04	6.61 ± 0.39	4.23 ± 0.51
0.5	6.57 ± 0.37	0.65 ± 0.01	6.60 ± 0.37	5.63 ± 0.31

Table S2 Dilatational properties of cyclohexane in water +0.01 g/L SDS

**Frequency (Hz)**	**E' (mN/m)**	**E" (mN/m)**	**E* (mN/m)**	**Phase Angle (°)**
0.1	9.76 ± 0.47	2.39 ± 0.35	10.06 ± 0.41	13.8 ± 2.40
0.2	11.1 ± 0.31	2.14 ± 0.90	11.3 ± 0.15	10.9 ± 4.75
0.3	10.4 ± 0.06	1.99 ± 0.16	10.6 ± 0.05	10.8 ± 0.89
0.4	10. ± 0.13	1.87 ± 0.28	10.2 ± 0.17	10.4 ± 1.45
0.5	10 ± 0.16	1.97 ± 0.06	10.2 ± 0.15	11.1 ± 0.46

Table S3 Dilatational properties of cyclohexane in water +0.1 g/L SDS

**Frequency (Hz)**	**E' (mN/m)**	**E" (mN/m)**	**E* (mN/m)**	**Phase Angle (°)**
0.1	9.19 ± 0.26	4.51 ± 0.52	10.2 ± 0.03	26.1 ± 3.25
0.2	11.7 ± 0.12	5.48 ± 0.02	12.9 ± 0.10	25.0 ± 0.31
0.3	13.1 ± 0.16	5.66 ± 0.02	14.2 ± 0.14	23.3 ± 0.33
0.4	13.6 ± 0.09	5.89 ± 0.02	14.9 ± 0.08	23.2 ± 0.23
0.5	14.7 ± 0.05	6.14 ± 0.06	15.9 ± 0.05	22.6 ± 0.20

Table S4 Dilatational properties of cyclohexane in water +1 g/L SDS

**Frequency (Hz)**	**E' (mN/m)**	**E" (mN/m)**	**E* (mN/m)**	**Phase Angle (°)**
0.1	7.02 ± 0.25	3.33 ± 0.05	7.77 ± 0.24	25.4 ± 0.48
0.2	8.81 ± 0.17	3.57 ± 0.02	9.50 ± 0.17	22 ± 0.28
0.3	9.74 ± 0.16	3.60 ± 0.05	10.3 ±0.17	20.3 ± 0.07
0.4	10.4 ± 0.18	3.57 ± 0.05	11 ± 0.18	18.9 ± 0.10
0.5	10.8 ± 0.15	3.53 ± 0.04	11.4 ± 0.16	17.9 ± 0.02

Table S5 Dilatational properties of PW+0.001 g/L SDS in cyclohexane

**Frequency (Hz)**	**E' (mN/m)**	**E" (mN/m)**	**E* (mN/m)**	**Phase Angle (°)**
0.1	10.7 ± 0.65	0.49 ± 0.22	10.7 ± 0.66	2.56 ± 1.04
0.2	13.3 ± 1.20	0.83 ± 0.24	13.3 ± 1.18	3.70 ± 1.43
0.3	11.4 ± 0.90	0.71 ± 0.05	11.4 ± 0.90	3.57 ± 0.14
0.4	12.2 ± 1.33	0.65 ± 0.33	12.2 ± 1.34	2.90 ± 1.26
0.5	12.7 ± 1.46	0.46 ± 0.35	12.7 ± 1.47	2.02 ± 1.45

Table S6 Dilatational properties of PW+0.01 g/L SDS in cyclohexane

**Frequency (Hz)**	**E' (mN/m)**	**E" (mN/m)**	**E* (mN/m)**	**Phase Angle (°)**
0.1	17.6 ± 0.08	8.77 ± 0.01	19.6 ± 0.07	26.4 ± 0.08
0.2	21.8 ± 0.11	9.78 ± 0.02	23.9 ± 0.10	24.1 ± 0.15
0.3	24.5 ± 0.09	10.2 ± 0.01	26.6 ± 0.08	22.7 ± 0.08
0.4	26.4± 0.06	10.5 ± 0.13	28.47 ± 0.09	21.7 ± 0.22
0.5	30.1 ± 2.84	11.2 ± 0.85	32.1 ± 2.96	20.3 ± 0.33

Table S7 Dilatational properties of PW+0.1 g/L SDS in cyclohexane

**Frequency (Hz)**	**E' (mN/m)**	**E" (mN/m)**	**E* (mN/m)**	**Phase Angle (°)**
0.1	4.71 ± 0.16	3.33 ± 0.07	5.77 ± 0.17	35.3 ± 0.42
0.2	6.11 ± 0.10	4.37 ± 0.02	7.5 ± 0.10	35.5 ± 0.31
0.3	7.22 ± 0.11	5.18 ± 0.04	8.8 ± 0.11	35.6 ± 0.24
0.4	8.38 ± 0.23	5.86 ± 0.10	10.2 ± 0.25	34.9 ± 0.26
0.5	9.75 ± 1.04	6.89 ± 0.81	11.9 ± 1.32	35.2 ± 0.28

Table S8 Dilatational properties of PW+1 g/L SDS in cyclohexane

**Frequency (Hz)**	**E' (mN/m)**	**E" (mN/m)**	**E* (mN/m)**	**Phase Angle (°)**
0.1	3.33 ± 0.24	2.39 ± 0.11	4.10 ± 0.26	35.6 ± 0.78
0.2	4.25 ± 0.28	3.37 ± 0.10	5.43 ± 0.28	38.4 ± 1.21
0.3	4.94 ± 0.28	4.15 ± 0.07	6.45 ± 0.22	40.0 ± 1.56
0.4	5.62 ± 0.40	4.77 ± 0.14	7.38 ± 0.39	40.3 ± 1.25
0.5	6.17 ± 0.26	5.38 ±0.14	8.19 ± 0.12	41.1 ± 1.91

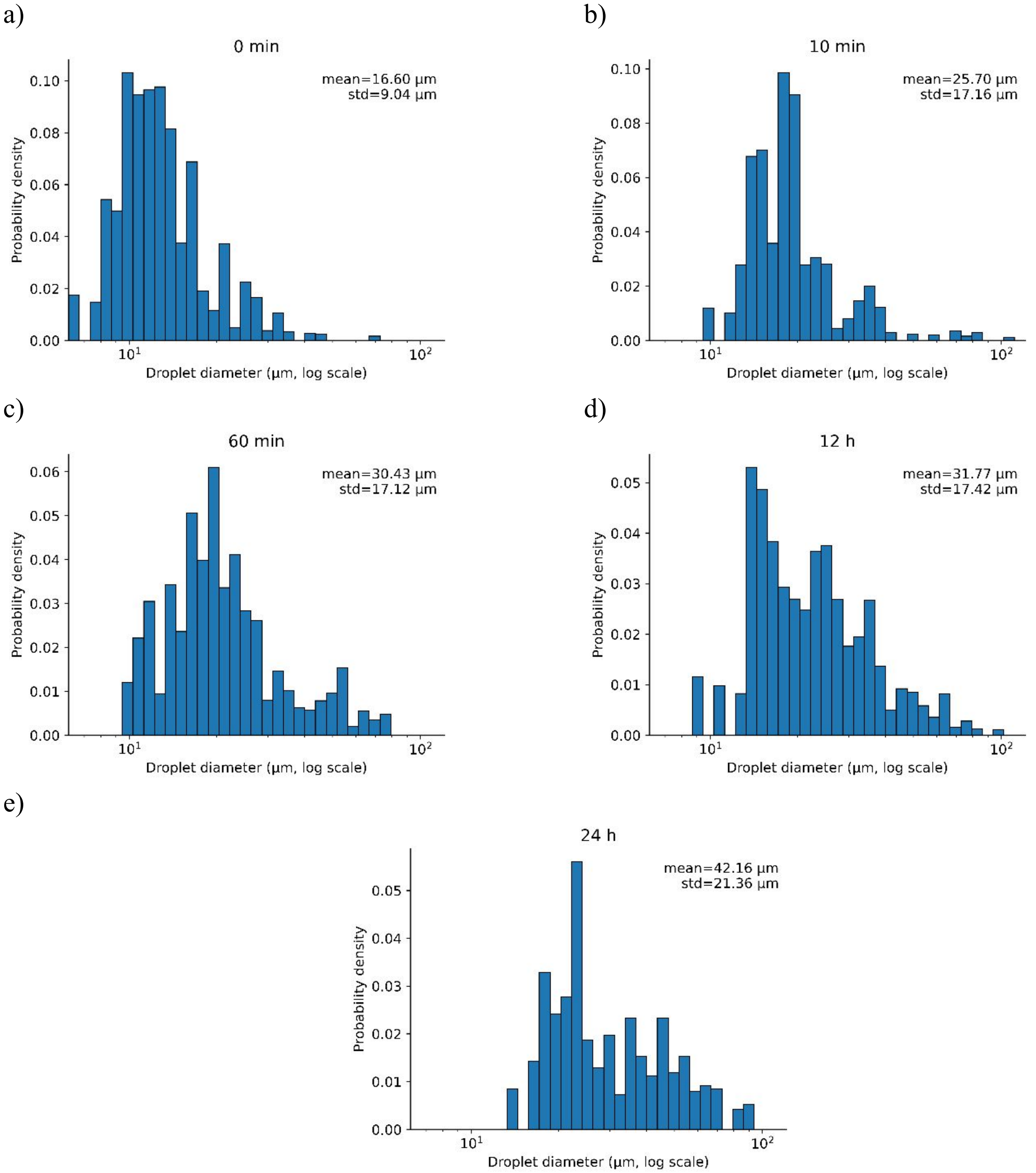

Figure S1. Probability density histograms of droplet diameter distributions for oil in PW emulsions containing 1 vol% cyclohexane in 99 vol% PW with 0.001 g/L SDS, measured at (a) 0 min, (b) 10 min, (c) 60 min, (d) 12 h, and (e) 24 h. The x-axis is shown on a logarithmic scale.

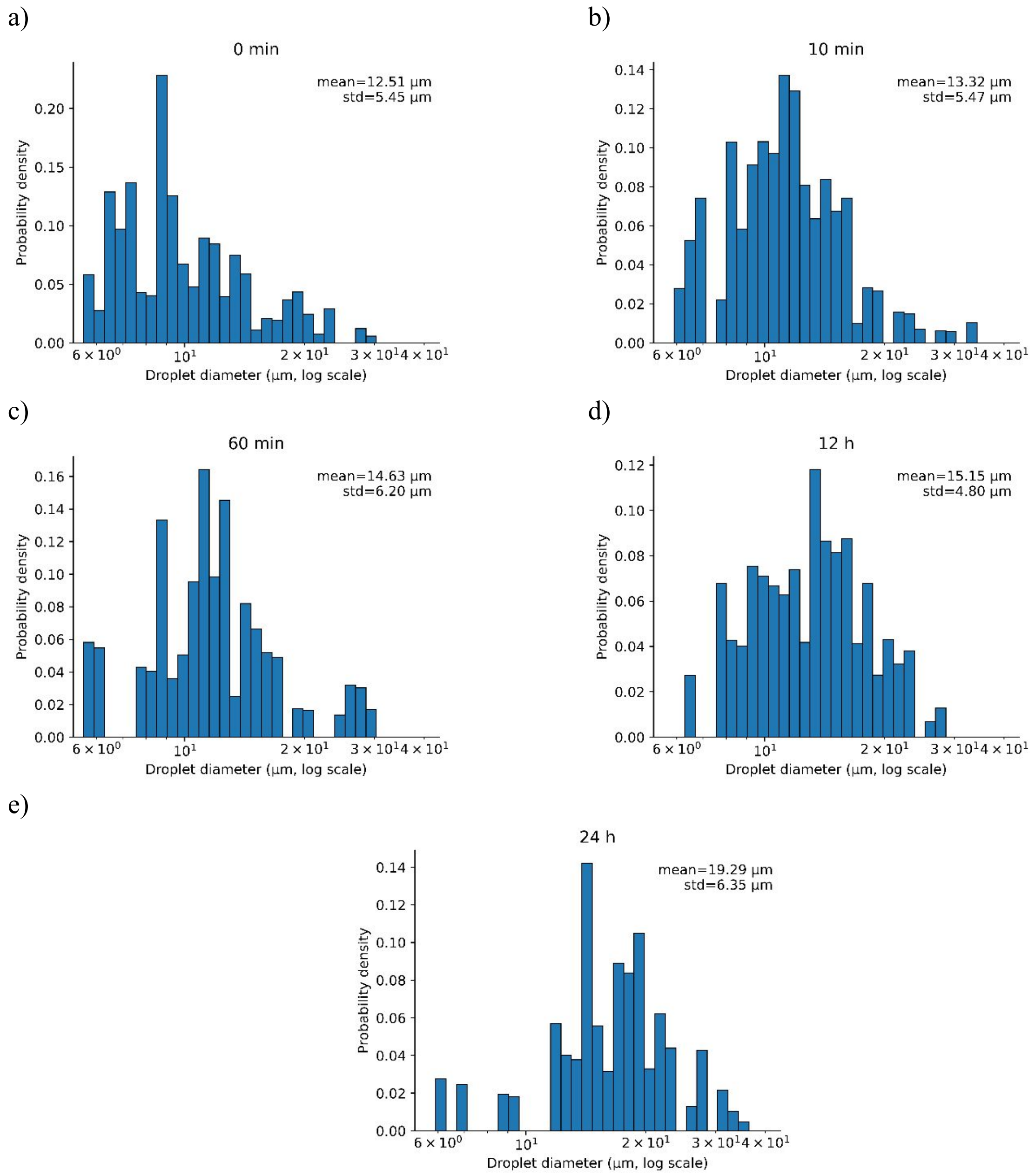

Figure S2. Probability density histograms of droplet diameter distributions for oil in PW emulsions containing 1 vol% cyclohexane in 99 vol% PW with 0.01 g/L SDS, measured at (a) 0 min, (b) 10 min, (c) 60 min, (d) 12 h, and (e) 24 h. The x-axis is shown on a logarithmic scale.

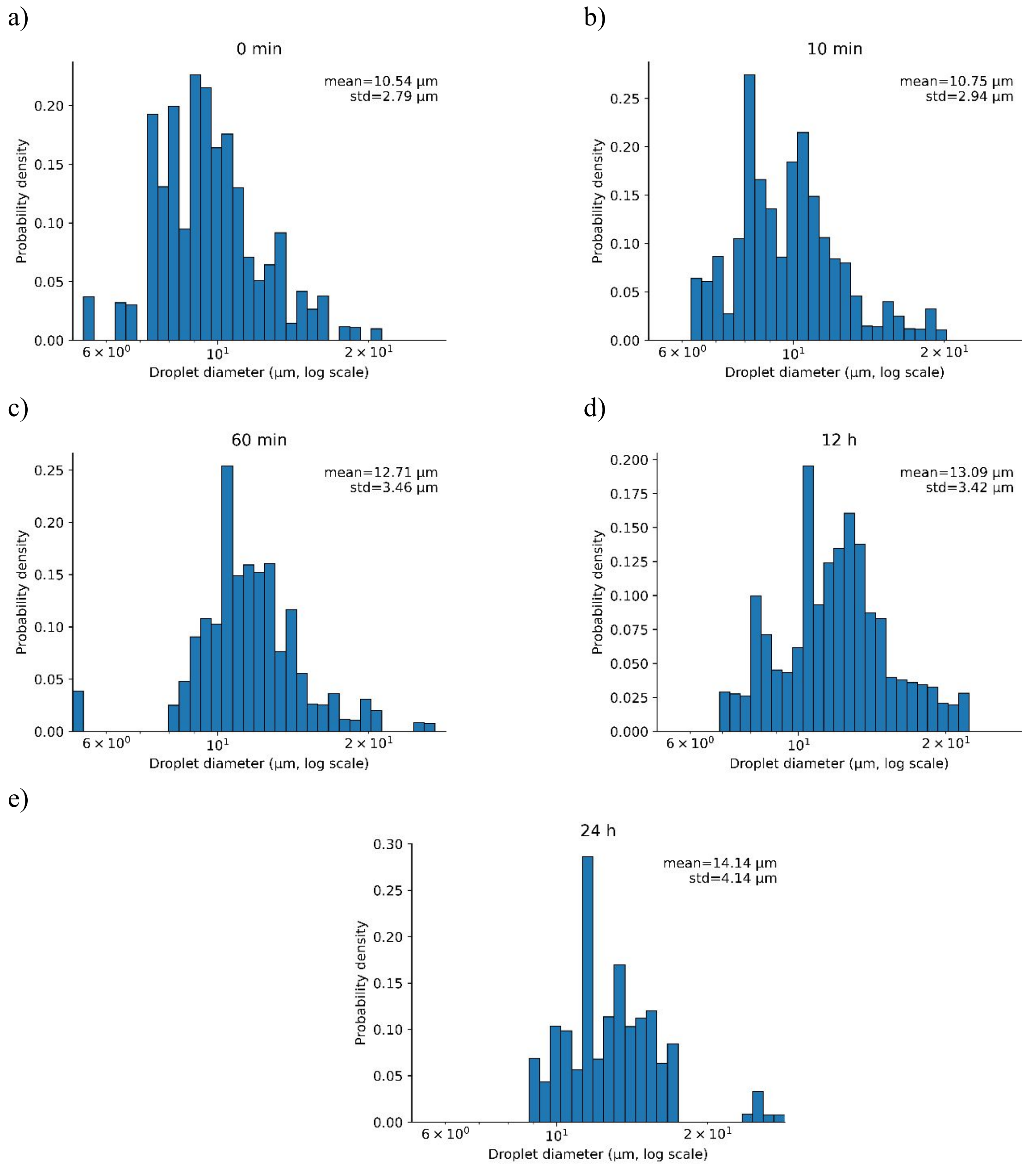

Figure S3. Probability density histograms of droplet diameter distributions for oil in PW emulsions containing 1 vol% cyclohexane in 99 vol% PW with 0.1 g/L SDS, measured at (a) 0 min, (b) 10 min, (c) 60 min, (d) 12 h, and (e) 24 h. The x-axis is shown on a logarithmic scale.

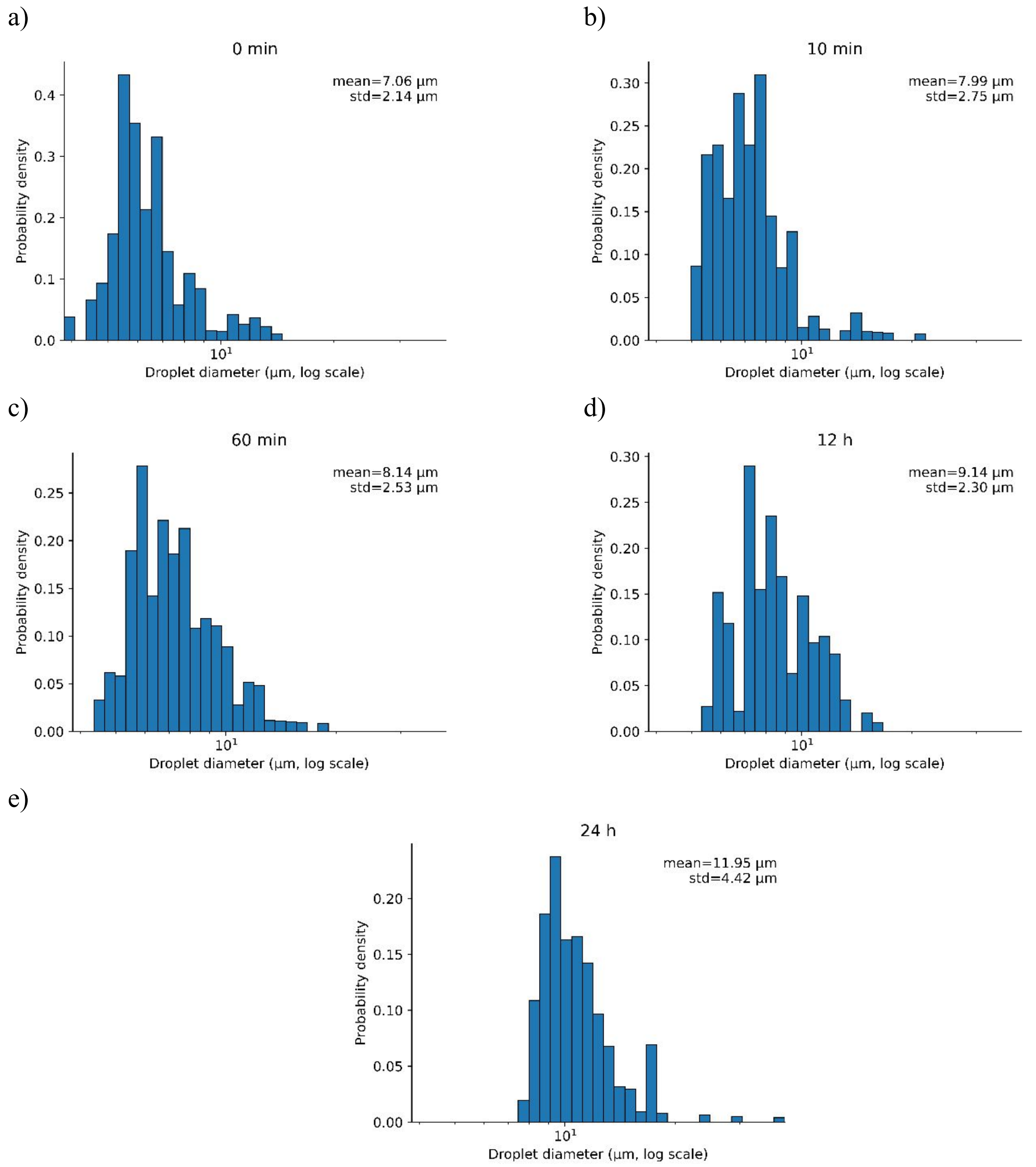

Figure S4. Probability density histograms of droplet diameter distributions for oil in PW emulsions containing 1 vol% cyclohexane in 99 vol% PW with 1 g/L SDS, measured at (a) 0 min, (b) 10 min, (c) 60 min, (d) 12 h, and (e) 24 h. The x-axis is shown on a logarithmic scale.
